# Genome-wide association study identifies a gene conferring high physiological phosphorus use efficiency in rice

**DOI:** 10.3389/fpls.2023.1153967

**Published:** 2023-03-14

**Authors:** Ming Yan, Fangjun Feng, Xiaoyan Xu, Peiqing Fan, Qiaojun Lou, Liang Chen, Anning Zhang, Lijun Luo, Hanwei Mei

**Affiliations:** ^1^ Shanghai Agrobiological Gene Center, Shanghai Academy of Agricultural Sciences, Shanghai, China; ^2^ Key Laboratory of Grain Crop Genetic Resources Evaluation and Utilization, Ministry of Agriculture and Rural Affairs, Shanghai, China

**Keywords:** GWAS, *OsAAD*, physiological phosphorus use efficiency, biomass, grain yields

## Abstract

Phosphate (Pi) is indispensable for the growth and development of plant, and low-Pi stress is a major limitation for crop growth and yield worldwide. The tolerance to low-Pi stress varied among rice germplasm resources. However, the mechanisms underlying the tolerance of rice to low-Pi stress, as a complex quantitative trait, are not clear. We performed a genome-wide association study (GWAS) through a diverse worldwide collection of 191 rice accessions in the field under normal-Pi and low-Pi supply in two years. Twenty and three significant association loci were identified for biomass and grain yield per plant under low-Pi supply respectively. The expression level of *OsAAD* as a candidate gene from a associated locus was significantly up-regulated after low-Pi stress treatment for five days and tended to return to normal levels after Pi re-supply in shoots. Suppression of *OsAAD* expression could improve the physiological phosphorus use efficiency (PPUE) and grain yields through affecting the expression of several genes associated with GA biosynthesis and metabolism. *OsAAD* would be a promising gene for increasing PPUE and grain yield in rice under normal- and low-Pi supply *via* genome editing.

## Introduction

Phosphorus (P) is one of the indispensable macronutrients for crops and is a major factor constraining the growth and development of crop worldwide in soil (Raghothama, 1999). Excess P fertilizers are applied to meet the high demands of rice due to the high P-fixing capacity of the soils (Vance et al., 2003). However, the non-renewable and finite reserves of phosphate resources would be exhausted not far in the future due to high inputs of P fertilizers. The low recovery rates of P fertilizers in season lead to increasing the cost of rice production and water eutrophication (Conley et al., 2009). Therefore, breeding and application of P-efficient crops would be a necessary strategy for sustainable crop production.

Phosphorus efficiency has typically been consist of P acquisition efficiency from native soil and internal P use efficiency (Veneklaas et al., 2012). The crops have developed many adaptive mechanisms to absorb P from soil, including increasing the number of root and change root architecture, secretion of organic acid to mobilization of sparingly soluble P in soil, the expression of genes related with Pi signal and uptake in roots, and symbiotic association with mycorrhizal fungi (Raghothama and Karthikeyan, 2005; Wu et al., 2013). Many genes related with rice Pi signal, uptake or translocation have been studied in detail. But overexpression or mutation of a Pi transporter gene or its regulatory gene often lead to excessive Pi accumulation with negative effects on plant growth, especially under Pi-replete conditions (Gu et al., 2015). A set of backcrossed inbred lines derived from a japonica x indica rice cross was used to identify a major quantitative trait locus (QTL) named *Pup1* on chromosome 12 of rice (Wissuwa et al., 2002). A protein kinase gene (*OsPSTOL1*) in the candidate region was cloned that enhanced P acquisition through increasing root growth in low-P soils (Gamuyao et al., 2012). QTL for P acquisition efficiency has received significant attention (Wang et al., 2010; Wu et al., 2013). However, physiological PUE has received less attention and QTLs are not available for breeding crops with enhanced PUE as it was difficult to measure (Rose et al., 2011). Improved PPUE may be realized by producing high yields of crops with lower P tissue concentrations to maintain the growth and development or by improving the efficiency of P remobilization from old organs to young, developing organs (Dissanayaka et al., 2018). Twenty six QTLs for PUE traits were detected in a two-year field experiment under normal and low P supply through a recombinant inbred population of rice derived from Minghui 63 and Zhenshan 97 (Wang et al., 2014). PUE loci were mapped on chromosomes 1, 4, 11 and 12 by using a rice germplasm panel of 292 genotypes of diverse plant types and origins that include all five subpopulations of *Oryza sativa* under hydroponic system that assured equal plant P uptake (Wissuwa et al., 2015).

Advances in sequencing technologies have made it possible to characterize the genetic variation presented in an increasing number of crop gene bank accessions. Genome wide association studies (GWAS) can investigate the populations with wide range of natural variation, and discovery larger numbers of important loci, especially for complicated agronomic traits (Huang et al., 2010; Zhao et al., 2011). In present study, we conducted GWAS using a diverse worldwide collection of 191 rice accessions which were re-sequenced on the Illumina HiSeq 2000 (Wu et al., 2015). Twenty loci associated with biomass and 3 loci associated with grain yield at the ripening stage were identified in the diverse germplasm panel that was grown under P deficiency condition in the field for two years. Candidate genes underlying significant associated regions were further analyzed. Six known genes which were responsible for Pi uptake, translocation, signaling and homeostasis were identified. We also discovered a new gene, *OsAAD*, encoding an amino acid dehydrogenase family protein. *OsAAD* enhances physiological PUE and grain yield by adjusting rice tillering. Thus, *OsAAD* turns out to be a promising gene for increasing the yield in rice under low and normal Pi supply *via* genome editing or marker-assisted breeding.

## Materials and methods

### Plant material and field experiment

Two subsets of rice germplasm resource described in detail by Wu et al. (2015) were used in this study. After excluding varieties with too short or too long growth periods, 191 accessions were included (**Supplementary Table 1**). The field experiments were carried out in Hainan experimental station of Shanghai Academy of Agricultural Sciences (SAAS) in Lingshui, Hainan, China (18°33′ N, 110°04′ E) in 2014 and 2015. Each line was transplanted with a spacing of 20 cm × 20 cm to plots with an area of 0.72 m^2^. Each plot included 3 rows with 7 hills per row. The plots were arranged following a randomized complete block design with four replicates. The total P concentration of the soil was 0.18 mg/g. Pi-fertilizer application amount was 26 kg P_2_O_5_/ha for low P treatment and 105 kg P_2_O_5_/ha for normal P treatment, respectively. All the Pi-fertilizer was applied as basal fertilizer in the form of calcium superphosphate. To achieve high grain yield, a total of 210 kg N ha^−1^ in the form of urea was applied twice: 105 kg ha^−1^ as basal fertilizer, 105 kg ha^−1^ 20 days after transplanting. Potassium sulfate (100 kg K ha^−1^) and zinc sulfate heptahydrate (5 kg Zn ha^−1^) were applied as basal fertilizer. A flood-irrigation system was adopted, which followed high-yielding agricultural practices. At the maturity of the plants from each plot, three uniform plants were sampled. Biomass per plant (BY) and grain yield per plant (GY) were measured.

### Genome wide association studies

The GWAS was conducted by using the CMLM algorithm implemented in GAPIT R package (Zhang et al., 2010). A total of 3,038,555 SNP markers were used for GWAS. The genome-wide threshold was first set at false discovery rate (FDR) = 0.05, and then it was set at *p* = 3.29E-07, calculated *via* the formula: 1/total number of SNPs, which was widely used in plant GWAS studies (Wen et al., 2014; Wang et al., 2015; Bai et al., 2016). We furthermore evaluated the extent of local linkage disequilibrium (LD) for each significant SNP. The extended region, where LD between nearly SNPs and lead SNP (with the lowest p-value) decayed to *r^2^* = 0.6, was defined as the local LD-based QTL interval (Yano et al., 2016).

### RNA extraction, cDNA synthesis and RT-qPCR

Total RNA was extracted using RNAiso Plus (TaKaRa) according to the manufacturer’s instructions. First-strand cDNAs were synthesized from total RNA using *EasyScript* ^®^ One-Step gDNA Removal and cDNA Synthesis SuperMix (TransGen Biotech). Reverse transcription quantitative PCR was performed with the PerfectStart Green qPCR SuperMix (TransGen Biotech) on the CFX96™ Real-Time System (BIO-RAD, USA). The relative level of expression was calculated by the equation 2^-ΔΔCt^ using housekeeping gene *OsActin1* (LOC_Os03g50885) as an internal reference (Feng et al., 2017). The primers used are listed in **Supplementary Table 2**.

### Production and identification of *aad* mutant lines

For the generation of *aad* mutant, the target sequence (**Supplementary Table 2**) was synthesized and ligated with respective sgRNA catastases and then were sequentially cloned into the CRISPR/Cas9 binary vectors pYLCRISPR/Cas9Pubi-H as previously described (Ma et al., 2015). The DNA sequence of *OsAAD* was amplified by PCR using gene specific primers (**Supplementary Table 2**), and PCR products were sequenced and aligned with wild-type sequences. Two homozygous knockout mutants (*aad-6* and *aad-7)* with different mutation sites were identified and used for subsequent physiological analysis (**Supplementary Figure 1**).

For hydroponic experiments, seeds were surface sterilized with 10% (v: v) H_2_O_2_ for 30 min, and rinsed thoroughly with deionized water. The sterilized seeds were germinated on PCR plate mounted in plastic containers for 1 week. The seedlings were transferred to a half-strength Kimura B solution with pH 5.6 (Yamaji et al., 2013) and grown in artificial climate chamber with a 14 h: 10 h as light: dark photoperiod and 30°C: 22°C as day: night temperature, and the relative humidity was controlled at *c.* 60%.

Pot experiments were conducted with four replications in artificial climate chamber using the soil collected from Hainan experimental station of SAAS. The acid soil contained 6.5 mg Pi kg^-1^ extracted by the Bray I method (Bray and Kurtz, 1945). One wild-type plant and one *aad* mutants were grown in each pot containing 6 kg of air-dried soil. The Pi fertilizer were 40 and 160 mg Pi kg^-1^ soil as low and normal supply levels, respectively.

### Measurement of total P concentration

For total P measurement, the shoots and roots of rice plants were dried to a constant weight at 70℃. Dried samples (0.5 g) were pre-digested in 100 ml glass tubes with 5 ml concentrated sulfuric acid (H_2_SO_4_) for 2 h. The tubes were then heated to 280℃ for 30 min, and 50 μl H_2_O_2_ was added every 10 min until the solution became colorless. The digestion was continued for another 30 min. The dilution of the digested solution was analyzed by the molybdenum blue method according to the procedure of Ames (1966).

### Definition of PPUE

The P concentration of shoot and root is the P accumulation divided by its corresponding dry weight. The physiological phosphorus use efficiency (PPUE) is tissue dry weight divided by its P concentration under low or normal-Pi supply (g^2^ DW g^-1^P).

### RNA-seq analysis

Four-leaf-old seedlings of WT, *aad-6* and *aad-7* mutants grown under low-Pi supply for two weeks were harvested for subsequent RNA-seq analysis. Total RNA was extracted from the shoots and roots of three biological replicates (5 seedlings per replicate) using RNAiso Plus (TaKaRa). RNA-seq library was constructed with the TruSeq RNA Sample Preparation v2 Guide, and RNA sequencing was performed using Illumina HiSeq 2500 by Shanghai Personal Biotechnology Co., Ltd. After filtering adapters and low-quality reads, the paired-end reads were then aligned to the reference genome of rice using HISAT2 v2.1.0 (Kim et al., 2019). Fragments per kilobase per million mapped (FPKM) reads was then used to estimate the expression level of the genes. The genes with the parameter of false discovery rate (FDR) below 0.01 was considered as differentially expressed genes (DEGs). Function of the DEG was predicted using Kyoto Encyclopedia of Genes and Genomes (KEGG, https://www.genome.jp/kegg/) databases (Xie et al., 2011). The raw reads were deposited in the National Center for Biotechnology Information Gene Expression Omnibus (NCBI GEO) with the accession number of PRJNA306542.

## Results

### Statistics analysis of phenotypic traits

Two traits varied widely among genotypes under both normal and low Pi supply in the field in two years. Variation for biomass in the P-deficient plot and P-replete plot ranged from 2.30 g to 18.30 g and from 3.93 g to 28.17 g in two years, respectively. Variation for grain yield in the P-deficient plot and P-replete plot ranged from 1.30 g to 8.35 g and from 2.87 g to 17.48 g in two years, respectively (**Table 1**). Under P-deficiency, the phenotypic mean values of the population were seriously reduced. P deficiency reduced biomass accumulation in Pi-deficient plots by 60.49% and 60.72% in 2014 and 2015, respectively. P deficiency reduced grain yield in Pi-deficient plots by 61.63% and 66.76% in 2014 and 2015 (**Table 1**; **Supplementary Figure 2**). The ANOVA results revealed significant effects from genotypes and treatments on both traits (**Table 2**). The high phenotypic ranges in this natural population indicated that these germplasm accessions contained rich genetic variations related to P-efficiency.

**Table 1:** Descriptive statistical results for traits under normal and low Pi supply in the natural population in the field experiments conducted in 2014 and 2015.

Trait	Year	Treatment	Mean	SD	CV (%)	Range
BY	2014	NP	11.49	3.11	27.07	5.55-23.66
		LP	4.54	2.02	44.52	1.43-13.40
	2015	NP	18.05	5.80	32.13	4.82-33.78
		LP	7.09	4.01	56.61	1.42-23.48
	Two years	NP	14.72	3.93	26.70	3.93-28.17
		LP	5.78	2.68	46.37	2.30-18.30
GY	2014	NP	10.45	2.98	28.54	3.33-18.45
		LP	4.01	1.24	31.02	0.92-7.58
	2015	NP	11.07	3.80	34.27	2.00-20.42
		LP	3.68	1.96	53.19	0.35-9.70
	Two years	NP	10.75	2.87	26.70	2.87-17.48
		LP	3.83	1.30	33.94	1.30-8.35

BY, biomass per plant (g/plant); GY, grain yield per plant (g/plant); CV, coefficient of variation.

**Table 2:** ANOVA of traits based on data from experiments in two years.

Trait	Variation	SS	df	MS	*F*	*P*-value
BY	Genotype	7204.858	189	38.121	1.857	2.06E-07
	Treatment	15220.012	1	15220.012	741.374	2.55E-91
	Genotype × Treatment	1307.940	189	6.920	0.337	1.00E+00
	Error	7801.198	380	20.529		
GY	Genotype	2600.220	189	13.758	2.976	1.07E-19
	Treatment	9070.619	1	9070.619	1961.955	3.88E-152
	Genotype × Treatment	1160.163	189	6.138	1.328	1.08E-02
	Error	1756.837	380	4.623		

SS, sum of squares; df, degree of freedom; MS, mean square.

### Genome-wide association study

**Figure 1:**
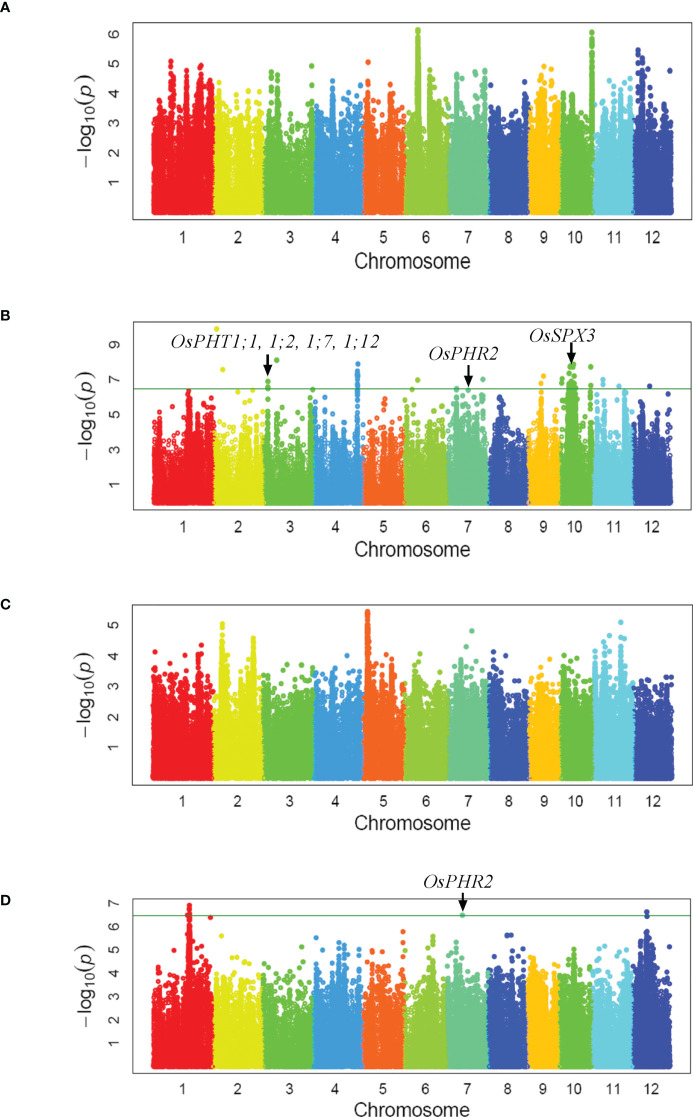
Genome-wide association study. Manhattan plots of GWAS mapping for two-year averages of biomass per plant **(A, B)** and grain yield per plant **(C, D)** measured under normal **(A, C)** and low Pi-fertilizer supply **(B, D)**, respectively.

As the population was used in GWAS of mesocotyl elongation, a two-subpopulation structure corresponding to *indica* and *japonica* subspecies has been reported in our previous publication (Wu et al., 2015). Using 3,038,555 SNPs covering the entire rice genome, a total of 20 loci associated with biomass per plant under low-Pi supply were identified under a Compressed Mixed Linear Model. Those associated loci were located on chromosome 1, 2, 3, 4, 6, 7, 9, 10, 11 and 12 (**Figure 1B**, **Supplementary Table 3**). However the significant loci associated with biomass per plant under normal-Pi supply was not detected (**Figure 1A**). Four Pi uptake genes (*OsPHT1;1, 1;2, 1;7, 1;12*), Pi signaling gene (*OsPHR2)* and Pi homeostasis gene (*OsSPX3*) were located within three QTLs intervals (Zhou et al., 2008; Ai et al., 2009; Sun et al., 2012; Dai et al., 2022; **Figure 1B**). Only three loci on chromosome 1, 7 and 12 associated with grain yield per plant under low-Pi supply were detected, but none of loci associated with grain yield per plant under normal-Pi supply was identified (**Figures 1C, D**). We found that *qGY7* and *qBY7.2* were in the same QTLs intervals which contained *OsPHR2* regulating Pi uptake (**Figures 1B, D**; Zhou et al., 2008). The candidate genes from 23 GWAS loci associated with biomass and grain yield per plant (QTL interval, *r^2^* of LD > 0.6) were further screened according to their expression levels in response to phosphate starvation and recovery (Secco et al., 2013). Among them, the expression levels of four genes were significantly up-regulated after 7-day Pi starvation and then tended to return to normal levels after Pi re-supply (**Supplementary Table 4**). These results indicate that these four genes might be good candidates for the GWAS loci. All four genes were knockdown by CRISPR/Cas9 in the Nipponbare, but only the phenotype of *aad* (LOC_Os09g15810) mutants was different from wild-type plants. The function of *OsAAD* in response to low-Pi stress then was investigated in detail.

### Expression patterns of *OsAAD* is responsive to low Pi

**Figure 2:**
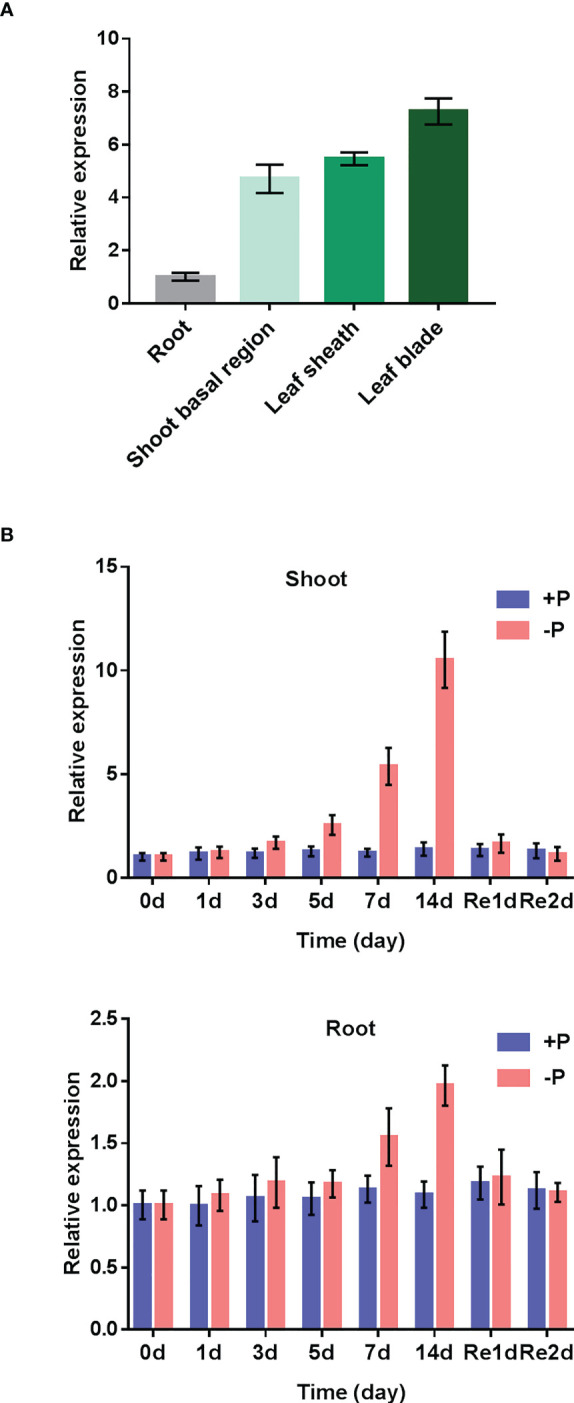
Expression patterns of *OsAAD* gene. **(A)** Gene expression in roots, shoot basal region, leaf sheath and leaf blade that were separately sampled at the six-leaf-old stage under normal Pi supply. **(B)** Four-leaf-old seedlings transferred into nutrient solution supplied with normal (200 m M Pi) or low (10 m M Pi) for 14 d, and then resupplied with normal Pi for 2 d. The shoots and roots were sampled at the beginning (0 d) and 1, 3, 5, 7 and 14 d after the treatment, and 1 and 2 d after the resupply of Pi. Values represent means ± SD of three biological replicates.

To check the spatial expression patterns of *OsAAD* in rice, reverse transcription quantitative (RT-qPCR) analysis was conducted for roots, shoot basal region, leaf sheath and leaf blade of seedlings supplied with normal Pi. As shown in **Figure 2A**, *OsAAD* was much more abundant in leaf blade than in other parts. A time-course treatment was carried out to check the transcriptional expression of *OsAAD* in response to low Pi stress. In shoots, *OsAAD* was significantly induced by low Pi treatment from day 5 to 14. Resupply of Pi for 1-day inhibited the expression of *OsAAD* to a level comparable with normal Pi supply (**Figure 2B**). In roots, the moderate induction of *OsAAD* expression was only observed after 7-day low Pi treatment that was completely abolished by 1-day normal Pi resupply (**Figure 2B**).

### Knockdown *OsAAD* enhanced PPUE of shoot and root

**Figure 3:**
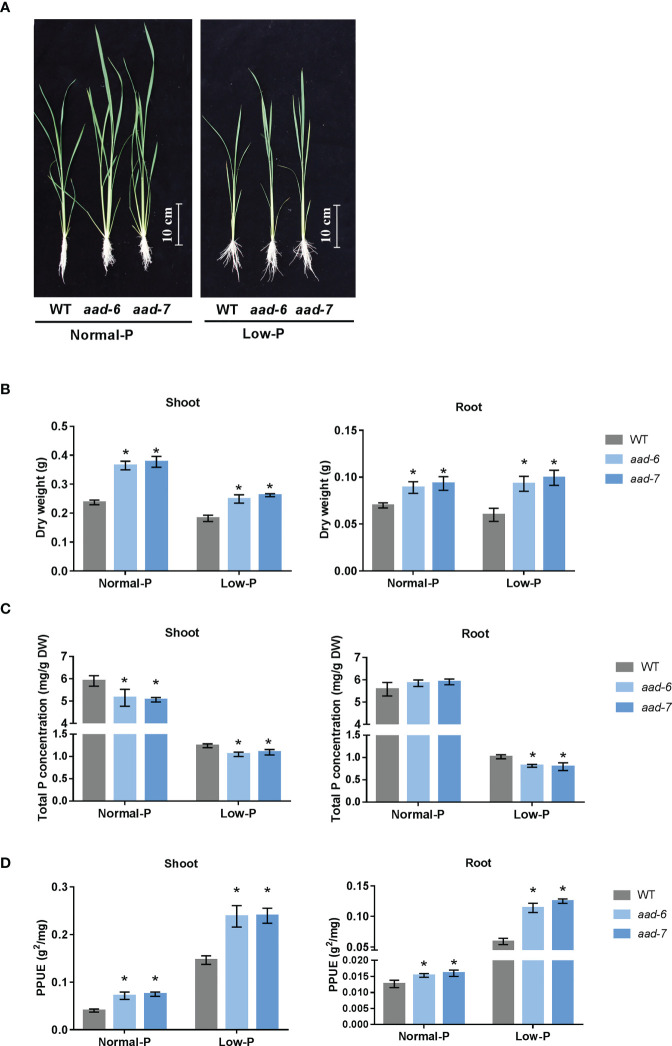
Phenotypic characteristics and total P concentration analysis of wild-types and *aad* mutants. **(A)** Growth performance of wild-type and *aad* mutants grown in a solution culture with normal Pi (200 m M) or low Pi (10 m M) supply for 30d. **(B)** Shoot and root dry weight of wild-type and *aad* mutants. **(C)** Total P concentration in shoot and root. **(D)** Physiological phosphorous use efficiency. Values represent means ± SD of three biological replicates *P < 0.01.

To investigate the physiological role of *OsAAD* in rice plants, corresponding mutant plants were obtained using the CRISPR-Cas9 system. Two independent lines (*aad-6* and *aad-7*) were used in further studies (**Supplementary Figure 1**). One-week-old *aad* mutants and wild-type seedling were cultivated in the hydroponic solution containing a normal and low level of Pi for 30 d. Two lines of *aad* mutants showed better growth than wild-type plants under both normal and low Pi supply (**Figure 3A**). The shoot and root dry weight of two *aad* mutants was significantly higher than wild-type plants under both normal and low Pi supply (**Figure 3B**). Total Pi concentration in shoots and roots of two *aad* mutants was lower than wild-type plants when grown in both normal and low Pi solution, except no difference in roots under normal Pi supply (**Figure 3C**). The PPUE of shoot and root in two *aad* mutants was higher than in the wild-type plants incubated in both normal and low Pi solution (**Figure 3D**). This suggested that *aad* mutants with lower tissue P concentrations could use less P-fertilizer to maintain the growth and development of rice.

### Mutation of *OsAAD* increases effective tiller number and grain yield

**Figure 4:**
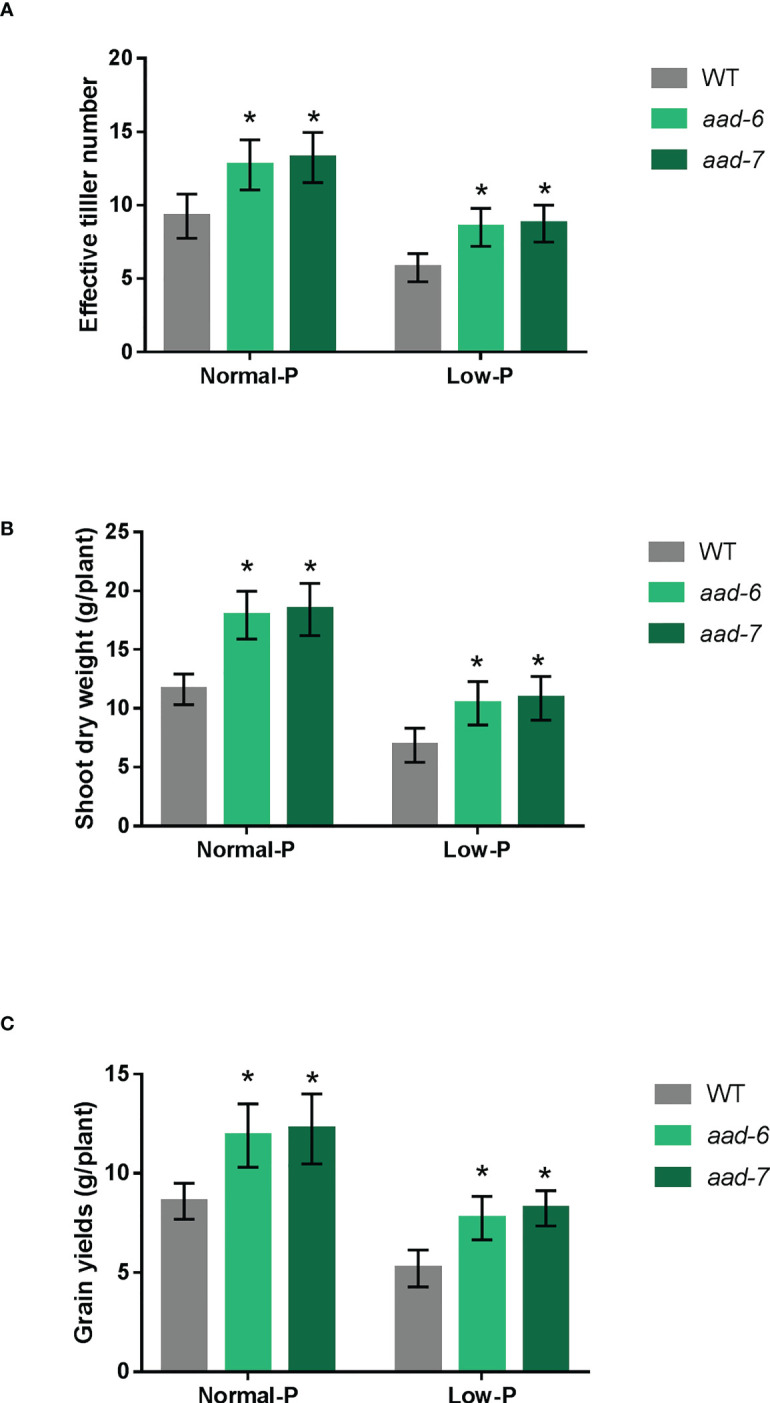
Growth performances of the wild-type and *aad* mutants under normal and low Pi supply in a pot experiment. **(A)** Effective tiller number per plant. **(B)** Shoot dry weight per plant. **(C)** Grain yields per plant. Values represent means ± SD of four biological replicates *P < 0.01.

To determine the function of *OsAAD* during the entire plant growth period, soil pot experiments were conducted in artificial climate chamber. We observed that two lines of *aad* mutants grew better than wild-type at 40 and 160 mg fertilizer Pi kg^-1^ soil. The effective tiller number, shoot dry weight per plant and grain yield per plant were measured at the harvest stage. The effective tiller number, biomass and grain yield were higher in *aad* mutants than in the wild-type plants under both Pi-deficient and Pi-replete soil (**Figure 4**). Furthermore, the grain yields of *aad* mutants under low Pi fertilizer treatment was almost equal to that of the wild-type under Pi-replete conditions (**Figure 4C**). Taken together, these results indicate that *OsAAD* would be a promising candidate gene for breeding rice with high P-efficiency under normal and low Pi supply.

### The *OsAAD* affects several genes involved in maintaining rice tillering

**Figure 5:**
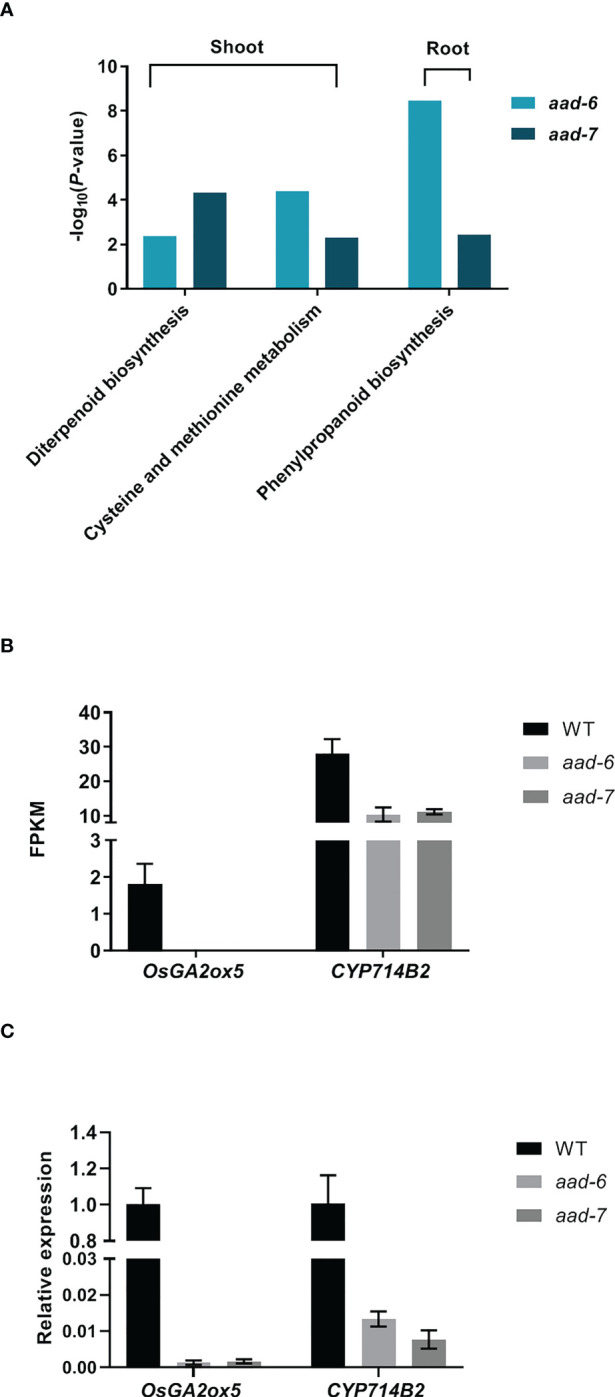
Transcriptome analysis of shoots and roots in two lines of *aad* mutants under low-Pi treatment for 2 weeks. **(A)** Histogram of the Kyoto Encyclopedia of Genes and Genomes (KEGG) pathway enrichment results. The X-axis is the name of the pathways, and the Y-axis is -log_10_ (*p*-value) enriched by each pathway. **(B)** and **(C)** FPKM and relative expression level of two genes related with GA pathway in shoots of two *aad* mutants and WT, respectively. Values represent means ± SD of three biological replicates.

To investigate the molecular mechanism underlying OsAAD-regulated rice tillering, a transcriptomic analysis was performed using the shoots and roots of two *aad* mutants and WT plants under low Pi condition for 2 weeks. Twenty and 18 genes were significantly upregulated in shoot and root, while 64 and 62 genes were downregulated in shoot and root, respectively, in two *aad* lines compared with the WT (**Supplementary Table 5**). Unexpectedly, 41 known genes associated with Pi uptake, translocation and signal were not differently expressed in *aad* mutants against the WT (**Supplementary Table 6**). Kyoto Encyclopedia for Genes and Genomes (KEGG) enrichment analysis revealed that diterpenoid biosynthesis, cysteine and methionine metabolism were significantly enriched in shoot, and phenylpropanoid biosynthesis was markedly enriched in root of both *aad-6* and *aad-7* mutants (**Figure 5A**). Among these differently expressed genes, the expression of *OsGA2ox5* gene which negatively regulate rice tiller was not detected, while the *CYP714B2* gene which is for GA metabolic enzymes was significantly downregulated in the shoots of two *aad* mutants (**Figure 5B**). The expression level of *OsGA2ox5* and *CYP714B2* in shoots of WT and two *aad* mutants was confirmed by qRT-PCR (**Figure 5C**). These results suggest that the *OsAAD* influenced tillering by the regulation of GA related gene expression.

### Natural variation of *OsAAD* might be tolerant to low Pi stress

**Figure 6:**
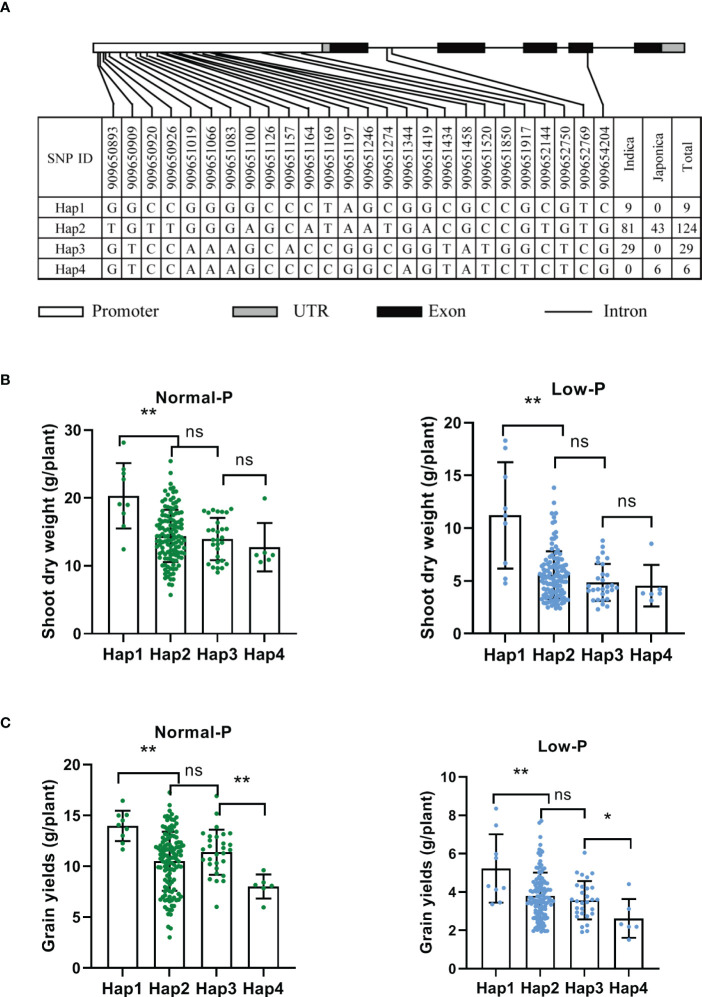
Haplotype analysis of *OsAAD*. **(A)** The gene structure of *OsAAD*. Four main haplotypes were detected by single nucleotide polymorphism (SNP) variations. The numbers of varieties in two subspecies of each haplotype are shown in the right column. **(B, C)** Shoot dry weight and grain yields per plant were compared among four haplotypes under normal and low Pi supply, respectively. All data are presented as the means ± SD. *P < 0.01; **P < 0.001, ns, not significant.

To uncover the natural variation of *OsAAD* and identify elite alleles, 191 rice varieties were used for haplotype analysis (**Supplementary Table 1**). Twenty-three SNPs were detected in 2k-bp promoter, two SNPs were located in the first intron, and only one SNP occurred in the exon of *OsAAD*. Based on these SNPs, four main haplotypes which contained at least five accessions were identified (**Figure 6A**). We found that hap1 and hap3 are belong to *indica* subgroup, while hap4 is a *japonic*-specific haplotype (**Figure 6A**). The shoot dry weight of hap1 was significantly higher than that of the other haplotypes under both normal and low Pi supply. Compared with normal Pi supply, the shoot dry weight of hap1 and hap2 under low Pi supply was decreased by 44.8% and 61.5%, respectively (**Figure 6B**). These results indicated that the tolerance of hap1 to low Pi stress was stronger than that of hap2. The grain yields of hap1 was also higher than that of the other haplotypes under both normal and low Pi supply. Among these four haplotypes, the grain yields of hap4 was lowest under both normal and low Pi treatment (**Figure 6C**). All these results suggested that hap1 would be a potential haplotype for breeding variety tolerant to low Pi stress through marker-assisted selection.

## Discussion

Tolerance to low-Pi stress is a complicated trait with low heritability and is strongly affected by both environment and genetic materials (Veneklaas et al., 2012). Based on reverse genetics, a lot of genes responsive for Pi signal and uptake were cloned and investigated in detail, but those genes were difficult to use for breeding P efficient variety in practical application (Gu et al., 2015). In this study, the tolerance to low-Pi stress of 191 rice germplasm accessions was evaluated under normal- and low-Pi supply in the Pi-deficit field in two years (**Supplementary Figure 2**; **Supplementary Table 1**). Twenty loci associated with biomass and three loci with grain yield under low-Pi supply was identified by using the average values measured in two years (**Figure 1**, **Supplementary Table 3**). One QTL (*qBY12*) on rice chromosome 12 located at the same region as the *Pup1* locus which was the most influential QTL mapped by using recombinant inbred line of Nipponbare and Kasalath in phosphorus-deficient soil (Wissuwa et al., 1998; **Supplementary Table 3**). It was subsequently shown to improve Pi uptake by promoting root growth (Gamuyao et al., 2012). Another QTL (*qBY3.1*) was mapped to chromosomal region containing four known P transporters (*PHT1;1, PHT1;2, PHT1;7 and PHT1;12*) of the PHT1 family which were in responsive for Pi uptake and translocation (**Figure 1**; **Supplementary Table 3**; Ai et al., 2009; Sun et al., 2012; Dai et al., 2022). Genes related to Pi signaling (*PHR2*) and Pi homeostasis (*SPX3*) were previously detected in QTL (*qBY7.2, qGY7*) and QTL (*qBY10.2*) interval, respectively (Zhou et al., 2008; Shi et al., 2014; **Figure 1**; **Supplementary Table 3**). All these results indicated that field experiments under Pi-deficit condition of diverse rice germplasm panel and GWAS for biomass and grain yield could serve as an efficient stretagy to identify genetic loci for tolerance to low P stress. These significant associated loci could be beneficial for understanding the molecular mechanism of the tolerance to low P and could be used for breeding new variety tolerant to low P stress through marker-assisted selection.

Via an integrative approach combining genomic mapping in this study and previous reported transcriptomic results (Secco et al., 2013), *OsAAD* was cloned as a potential candidate gene related to low-Pi stress tolerance. The expression level of *OsAAD* was induced by Pi starvation and tended to return to normal levels quickly after Pi re-supply in shoots (**Figure 2B**), in agreement with the reported RNA sequencing data (Secco et al., 2013). Growth performance of the wild-type, *aad-6* and *aad-7* mutants in nutrient hydroponic solutions and pot experiments under normal- and low-Pi supply showed that inhibition of *OsAAD* not only enhances shoot dry weight, effective tiller number and grain yield, but also increases physiological phosphorus use efficiency under normal- and low-Pi supply (**Figures 3** and **4**). The lateral root length of high PPUE cultivar was longer than that of low PPUE cultivar (Akhtar et al., 2008). The shoots and roots biomass of the *OsAVP1DOX* line grown in both Pi sufficient and Pi deficient conditions were higher than controls (Yang et al., 2007). We also found that the root dry weight of *aad-6* and *aad-7* mutants was higher than that of wild-type plants (**Figure 3B**). The total P concentration of shoot in two lines of *aad* mutants was lower than that of wild-type plants (**Figure 3C**). This suggested that *aad* muants were likely to use less Pi to maintain growth and tolerate to low Pi stress better. Compared with low-PUE genotype, high-PUE groups were able to reduce tissue P concentration to a lower level (Veneklaas et al., 2012; Adem et al., 2020). Meanwhile, significant changed expression level of other known genes related with Pi signaling, uptake, translocation and homeostasis was not detected in *aad* mutants (**Supplementary Table 6**). This indicated that low P concentration in the *aad* mutants was not due to the change of genes associated with Pi signaling, uptake, translocation and homeostasis.

The *OsAAD* encoded an amino acid dehydrogenase family protein according to genome annotation, consistent with the enriched cysteine and methionine metabolism pathway by KEGG analysis (**Figure 5A**). The substrate of OsAAD was still unknown and needed to be further explored in future. Diterpenoid biosynthesis was also significantly enriched in *aad* mutants by KEGG analysis (**Figure 5A**). The expression levels of *OsGA2ox5* gene which negatively regulate rice tiller numbers was not detectable, while the *CYP714B2* gene which is for GA metabolic enzymes was significantly downregulated in the leaf of two *aad* mutants (**Figure 5B**; Lo et al., 2008; Magome et al., 2013). This suggested that suppression of *OsAAD* expression increased tiller numbers by affecting the expression of genes related with GA biosynthesis and metabolism. Phosphorus played an important role in rice tillering which was inhibited by low available Pi in soil (Takehisa and Sato, 2019). The effective tiller numbers of *aad* mutants was much more than that of wild-type plants under normal and low Pi supply (Figuer 4A). The nucleotide diversity of *OsAAD* was characterized which revealed that hap1 was most tolerant to low P stress (**Figure 6**). The decrease of hap1 biomass caused by low P treatment was lower than that of other haplotypes. The germplasm accessions harbored elite hap1 in this study could be valuable for rice breeding. Taken together, *OsAAD* would be a promising gene for molecular breeding of rice cultivars with high yield and using less Pi to maintain the growth and development under low and normal Pi supply through genome editing.

## Data availability statement

The datasets presented in this study can be found in online repositories. The names of the repository/repositories and accession number(s) can be found below: BioProject, PRJNA306542.

## Author contributions

HM, LL and MY designed this study. MY, XX, QL, LC and AZ performed the field experiment. MY, HM and FF analyzed the data. MY and PF studied the function of candidate genes. MY and HM drafted the manuscript. All authors contributed to the article and approved the submitted version.
