# Supplementary material for: Genome-wide association study identifies a gene conferring high physiological phosphorus use efficiency in rice
Source: Front Plant Sci. 2023 Mar 14;14:1153967. doi: 10.3389/fpls.2023.1153967 (PMC10043302; doi:10.3389/fpls.2023.1153967)
Supplement: Supplementary file 1 [file DataSheet_1.pdf]

Supplementary Material

Genome-wide association study identifies a gene conferring high physiological phosphorus use efficiency in rice

Ming Yan†, Fangjun Feng†, Xiaoyan Xu, Peiqing Fan, Qiaojun Lou, Liang Chen, Anning Zhang, Lijun Luo* and Hanwei Mei*

*** Correspondence:** Corresponding Author: hmei@sagc.org.cn

Supplementary Figure 1. Identification of *aad* mutants. (A) Mutation type of *aad-6* and *aad-7*. The PAM (CCG) sites are highlighted in red. + and – indicated the nucleotide insertion and deletion, respectively. **(B)** Sequencing chromatograms of WT, *aad-6* and *aad-7* mutants.

(A)

WT	5’-TCCACTGCGACCATTA TTGATGG-3’
aad-6 (+T)	5’-TCCACTGCGACCATTATTTGATGG-3’
aad-7 (-A)	5’-TCCACTGCGACCATT- TTGATGG-3’

(B)

WT

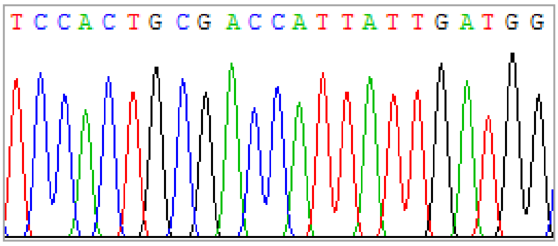

aad-6

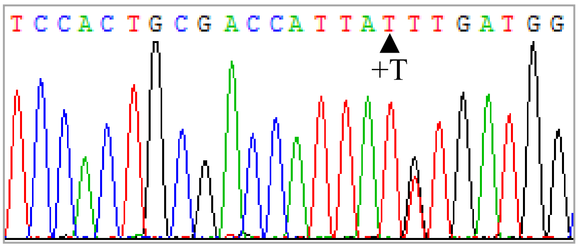

aad-7

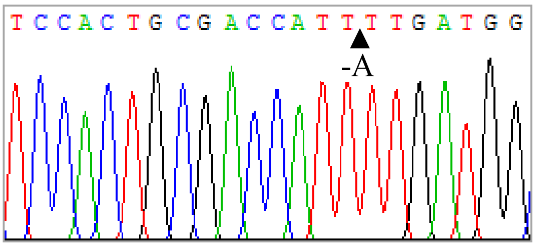

Supplementary Figure 2. The growth performance of the natural population in the fields under normal (A) and low (B) Pi-fertilizer supply.

(A)

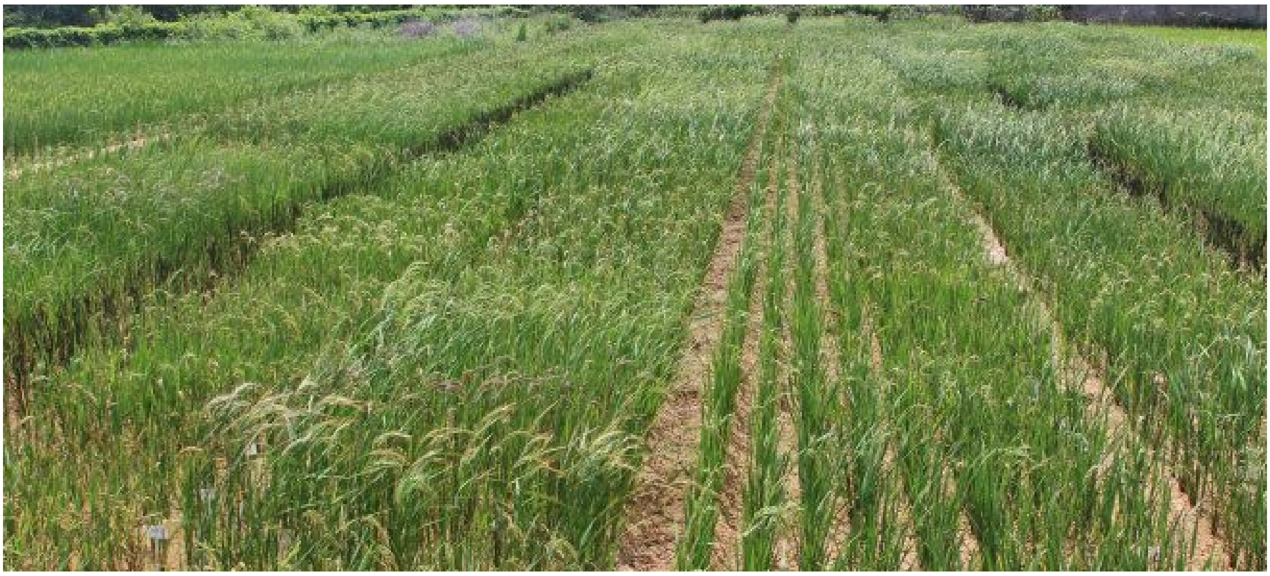

(B)

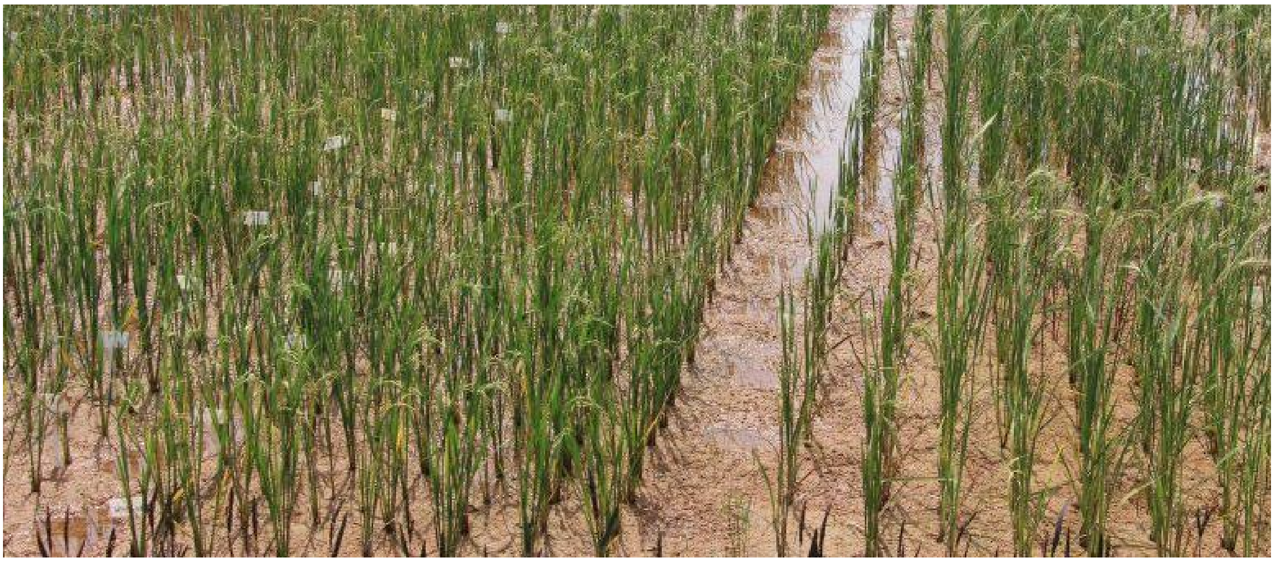

Supplementary Table 1. The data of biomass and grain yield in the natural population in the fields under normal and low Pi-fertilizer supply in two years.

Supplementary Table 2. All primers used in this study.

Supplementary Table 3. Summary of GWAS loci for biomass and grain yield per plant identified in this study.

Supplementary Table 4. The FPKM and fold changes of candidate genes in response to Pi stavation and re-supply in shoots.

Supplementary Table 5. The DEGs between WT and two lines of *aad* mutants in shoots and roots under low Pi stress.

Supplementary Table 6. The expression levels of known genes related with Pi uptake, signaling and homeostasis in shoots and roots of WT and two lines of *aad* mutants in response to low Pi stress.
